# AlphaFold3 at CASP16

**DOI:** 10.1002/prot.70044

**Published:** 2025-08-25

**Authors:** Arne Elofsson

**Affiliations:** ^1^ Department of Biochemistry and Biophysics and Science for Life Laboratory Stockholm University Stockholm Sweden

**Keywords:** AlphaFold, CASP, protein structure predictions, RNA structure prediction

## Abstract

The CASP16 experiment provided the first opportunity to benchmark AlphaFold3. In contrast to AlphaFold2, AlphaFold3 can predict the structure of non‐protein molecules. According to the benchmark presented by the developers, it is expected to perform slightly better than AlphaFold2 for proteins. In this study, we assess the performance of AlphaFold3 using both automatic server submissions (AF3‐server) and manual predictions from the Elofsson group (Elofsson). All predictions were generated via the AlphaFold3 web server, with manual interventions applied to large targets and ligands. Compared to AlphaFold2‐based methods, we found that AlphaFold3 performs slightly better for protein complexes. However, when massive sampling is applied to AlphaFold2, the difference disappears. It was also noted that, according to the official ranking from CASP, the AF3‐server performs better than AlphaFold2 for easier targets, but not for harder targets. Furthermore, the performance of the AF3‐server is comparable to the best methods when considering the top‐ranked predictions, but slightly behind when examining the best among the five submitted models. Here, there exist targets where AF3‐server, the top‐ranked method, is worse than lower‐ranked models, indicating that a venue for progress could be to develop better strategies for identifying the best out of the generated models. When using AF3‐server to predict the stoichiometry of larger protein complexes, the accuracy is limited, especially for heteromeric targets. When analyzing the predictions including nucleic acids, it was found that, in general, the accuracy is relatively low. However, the AF3‐server performance was not far behind that of the top‐ranked method. In summary, AF3‐server offers a user‐friendly tool that provides predictions comparable to state‐of‐the‐art methods in all categories of CASP.

## 1 Introduction

Protein structure prediction has been revolutionized in the last decade, starting with the rediscovery of DCA [1, 2] and other methods to predict contacts from multiple sequence alignments. It has been demonstrated that these methods could accurately predict the structure of proteins given sufficiently deep multiple sequence alignments [3, 4, 5]. These methods were then improved by utilizing various machine learning methods to post‐process these predictions [3, 6, 7, 8]. From the perspective of CASP, progress could first be seen at CASP13 when AlphaFold(v1) [9] outperformed other methods for challenging protein targets [10]. With the introduction of AlphaFold2.0 [11] at CASP14, the quality of predictions increased significantly.

In addition to predicting the structure of individual proteins, contact prediction methods can be used to predict the structure of protein complexes [12]. The so‐called Fold and Dock approaches showed some progress in CASP14 using machine learning– enhanced contact predictions [13]. With the release of AlphaFold2, these methods could suddenly predict the structure of many complexes accurately [14]. When these methods were applied in CASP15, it was confirmed that AlphaFold2 could accurately predict the structure of many complexes [15]. One challenge that seemed to remain was the prediction of AntiBodies/NanoBodies to Antigens, but increased sampling helped in some cases [16, 17].

However, proteins are not the only molecules in a cell; nucleotides, lipids, ions, and small molecules are also present. In 2024, several tools to predict the interactions between all these types of molecules were released [18, 19], and CASP16 was the first opportunity to benchmark these in a completely blind fashion. At the time of the CASP16 submissions, AlphaFold 3 was only available as a web server, limiting its usability. The main limitation was that only a few standardized protein ligands could be used; additionally, complexes larger than 5000 “tokens” could not be predicted.

For CASP16, I submitted manual predictions as group Elofsson (#241) and server predictions as AF3‐server (#304). All predictions were made using the AlphaFold3 server [1]. The five models from one target were submitted for the server predictions. In contrast, more models were generated, and good models with some structural variability were submitted for the manual predictions. For some models, additional changes were made; see the methods section.

## 2 Methods

We used the AlphaFold3 server for all predictions in CASP16, as installing it locally was impossible at that time. We had to make manual interventions to ensure it worked for all targets, but we tried to use it as automatically as possible. Manual interventions were necessary for ligand targets with unknown stoichiometry and targets larger than 5000 residues. Details on how this was achieved are provided below.

In addition to the automatic predictions from the AF3‐server (#304), we also submitted manual predictions from the Elofsson group (#241). The only difference for the Elofsson predictions was that we generated additional models by running the AF3‐server with various random seeds. We then used the internal AlphaFold3 scores and conducted manual inspections to select the models for submission. The results below indicate that manual intervention yielded no significant improvement; therefore, it will not be discussed in detail here.

### 2.1 Predictions

#### 2.1.1 Big Targets

Some targets (e.g., H1227, H1257) were too big to run on the server (the maximum is 5000 residues/nucleotides). For these targets, we used the strategy from MolPC [ 14], that is, building different types of overlapping fragments, superimposing the shared parts, and generating complete models. The cutting was not fully automated for the AF3‐server predictions, as we attempted to select the optimal location for the cut. However, for AF3‐server predictions, we only used one cut and submitted the top five models, while for the Elofsson predictions, we tried several versions. No refinements were done in any case.

#### 2.1.2 RNA

For multichain RNA molecules, it was noted that many models contained significant overlap. These can easily be detected from the scores provided by AF3‐server, as the flag “has_clash” is set and the “ranking_score” is negative. In total, 72% (309/420) of the multimeric RNA models we generated had such clashes and were therefore discarded. We excluded these whenever possible for submission. However, this necessitated additional submissions to the server for these targets until five non‐clashing models were obtained.

#### 2.1.3 Ligand Predictions

For ligand targets, it was not possible to use the exact ligand at the AF3 server. Therefore, we first manually identified the most similar (by number of carbon atoms primarily) ligand that was available. Thereafter, we ran the AlphaFold3 server with this ligand. Next, we generated a structural model from the SMILES using the https://www.novoprolabs.com/tools/smiles2pdb online tool. Finally, the CASP ligand was superposed on the AF3‐server model, generating a new PDB/Ligand file. Unfortunately, we submitted the ligands in PDB format without receiving any errors, so they were not officially evaluated by the prediction center (we provided them in the correct format later, but we assume that was too late).

#### 2.1.4 Ensemble Targets

Different strategies were used for different ensemble targets.

- R1203, T1214, R0283: Here we used standard predictions using the AF3‐server.
- T1294, T1200/T1300, M1239, T1249, M1228, T2249, R1253. Here, we generated many models and submitted ensembles of them after clustering.
- R1260 (solvation shell): For AF3, an ensemble of models was generated and hydrated using standard protocols. The Elofsson group took the best models and ran a 47‐ns‐long MD simulation.

### 2.2 Unknown Stoichiometry

For targets with unknown stoichiometry, we explored the possibility of predicting stoichiometry by creating models with different stoichiometries. Due to computational constraints, it was not feasible to generate all possible stoichiometries, particularly for heteromic targets. For homomers, we typically began with monomers and tried all stoichiometries up to hexamers. Since we did not investigate larger complexes, we were unable to determine the correct stoichiometry for larger targets, including R1253, an octamer, and thus failed to predict it accurately. Following the initial screening, we utilized the ranking confidence and selected the five best scores for AF3 submissions. Simultaneously, for the Elofsson submissions, we often chose a more diverse set of predictions, also considering manual inspection of the PAE maps.

For heteromeric targets containing multiple chains, that is, M and H targets, the number of possible stoichiometries was too large for a systematic search. Therefore, we only used a limited set of combinations. Starting from the assumption of one copy of each chain, we added individual copies of each chain until the ranking confidence decreased. As for homomers, we also evaluated the PAE maps for the Elofsson predictions.

### 2.3 Evaluations

CASP employs various measures to evaluate model accuracy. Each of these methods offers unique insights, but many of the measures are highly correlated with one another. A detailed analysis of all predictions using different measures will be presented elsewhere in this issue. Thus, our analysis will apply GDT_TS [20] for monomers, TMscore [21] for nucleic acids, and DockQ [22, 23] for complexes. We also present data regarding the ranking of the methods using *Z*‐score analysis from the prediction center, as this provides a direct comparison with all other prediction groups. All data is downloaded from the prediction center, and all analysis scripts are freely available from https://gitlab.com/arneelof/CASP16‐predictions. Except for stoichiometry analysis, we only analyzed Round 1 models, as we made no changes for Round 2, and the only alteration between Round 0 and Round 1 was a change in stoichiometry if necessary. In all pairwise comparisons, only the targets by both methods were included. Finally, only one result for H1265, which appears four times on the prediction centers website, was included; that is, H1265v1. H1265v2 and H1265v3 were ignored.

## 3 Results

In this paper, we analyze the performance of the AlphaFold3 server in CASP16. It should be remembered that all (manual) groups had access to the AF3‐server. Therefore, this can be seen as a baseline for all predictions. We primarily focused on comparing the performance of the AF3‐server in general to other methods and did not focus on the predicted models of individual targets. First, we compared the AF3‐server predictions with our manual predictions, which were based on running the server more extensively and manually selecting the best model. Secondly, for proteins, we compared the predictions of the AF3‐server with those of the baseline AlphaFold2 method, Colabfold_baseline. Finally, we compared the AF3‐server models with the best methods according to default scoring on the prediction center website. Comparisons are made separately for pure protein targets and targets that contain nucleic acids.

What is essential to consider is that this paper is not focused on replicating the excellent work of the assessors. Therefore, we use one standard measure, GDT_TS [20], for the quality of a single protein domain, TMscore [21] for nucleic acid structures, and another, DockQ [22, 23], for the quality of complexes. Indeed, a more detailed analysis could provide additional insights, but these measures are sufficient to detect significant differences between merit methods. If the differences are slight, the gap between the methods is minor. Furthermore, this is not a detailed analysis of individual predictions, as our predictions were made with minimal manual intervention. Therefore, we cannot always ascertain why one method performs better than another for a specific target; it often comes down to coincidences. As shown below, the reason why one method appears superior to another can usually be attributed to a small number of targets. This means that even if a difference is statistically significant, it may be of minimal importance for the average user if caused by one or a few targets, as these may be coincidences. Therefore, we disregard these and focus solely on systematic differences between methods.

### 3.1 Proteins

**FIGURE 1:**
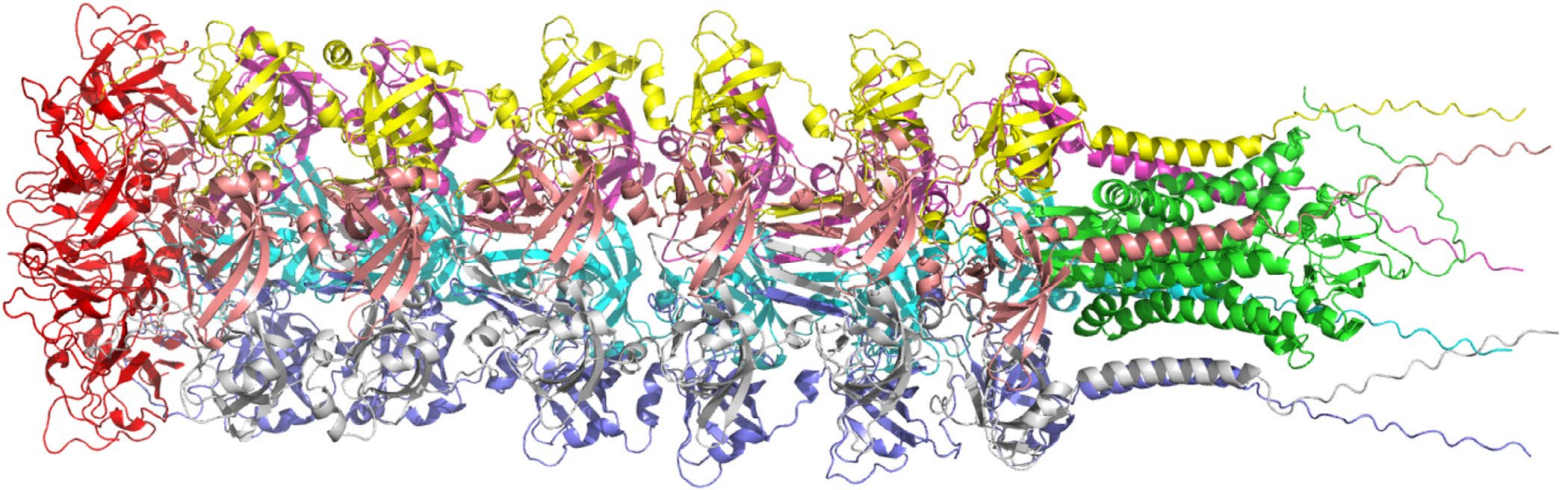
Modeling a large target, H1227, where the left domain (colored in red) could not be modeled directly by AF3‐server as the total complex was more than 5000 residues. Instead, it was modeled separately, along with the terminal part of the larger complex. The overlapping domain was superimposed, and the red domain was finally added to the model before submission.

All models were predicted using the AF3‐server default settings. However, if the target was bigger than 5000 residues, it had to be cut into pieces. In these cases, manual decisions were made on where to cut it. The pieces were then merged into a larger protein by superimposing the overlapping, as in MolPC [14]. One example of this methodology is illustrated in Figure 1.

#### 3.1.1 Elofsson Versus AF3

**FIGURE 2:**
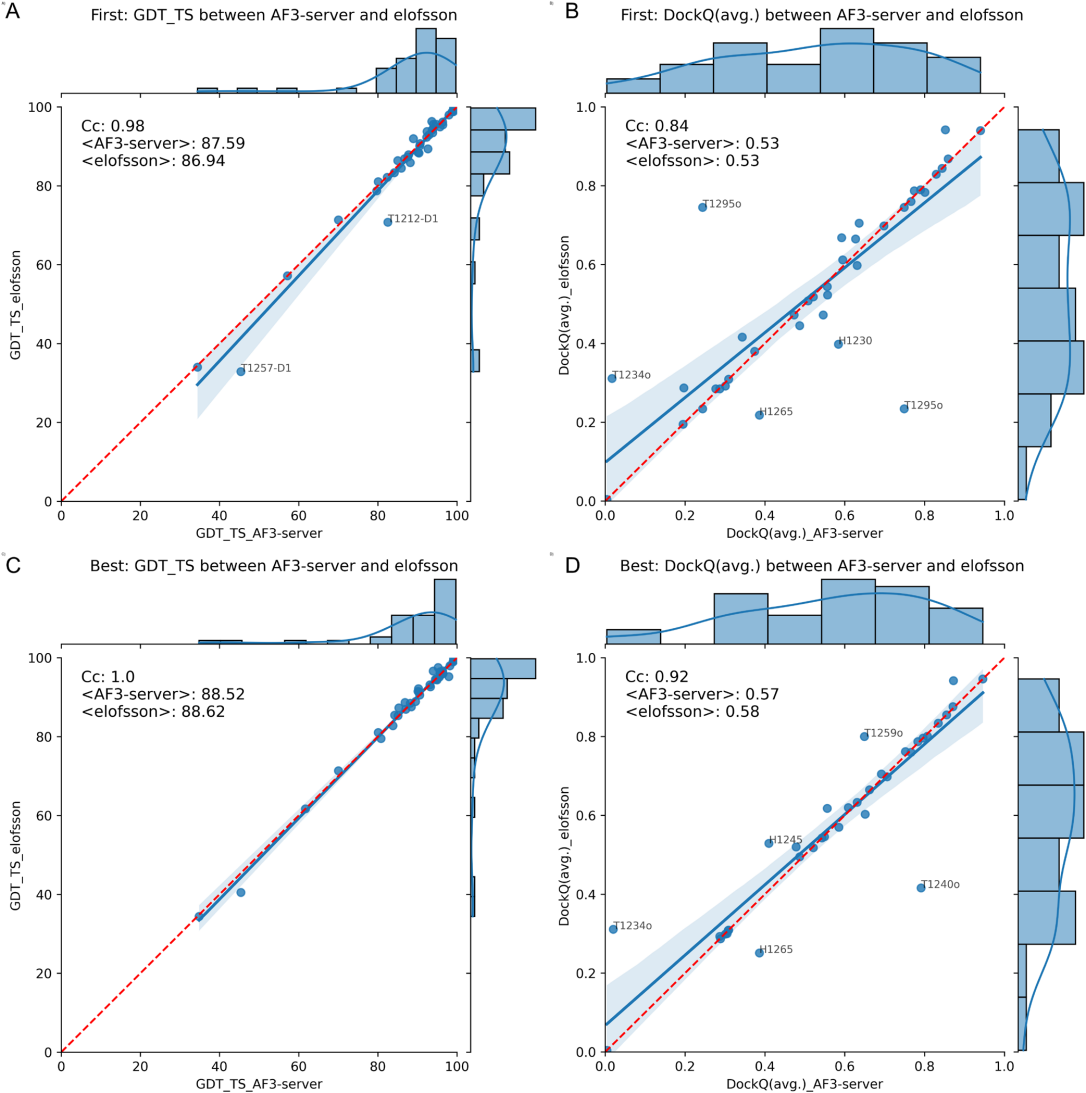
Comparison of AF3‐server and Elofsson predictions using GDT_TS and DockQ. The top row (A + B) compares the highest‐ranked prediction, while the second row (C + D) compares the best prediction out of the five submitted models. On the left (A + C), the comparisons are made at the domain level using GDT_TS, and on the right (B + D), comparisons of complexes using DockQ are shown. Any target with a difference greater than 10% of the maximum value for one of the methods is annotated with the target number.

We first explored whether our manual interventions, submitted as the group Elofsson, were effective. Figure 2 shows that the difference in model quality between AF3‐server and Elofsson is slight for the individual domains. When we examine the average GDT_TS scores (Figure 2A), AF3‐server shows slightly better performance. However, this difference originates from two domains: T1212‐D1 and T1257‐D1. When considering the best of the five predictions (Figure 2B), this difference disappears, indicating that, in these cases, AF3‐server happened to select a better first‐ranked model. It is also worth noting that all but four domains are predicted with high quality, as indicated by a GDT_TS score over 80. The average GDT_TS score is approximately 88, indicating a level of quality comparable to experimental results.

For the complexes (Figure 2B,D), there is no significant difference between AF3‐server and Elofsson predictions, nor when comparing the first‐ranked or the best of the submitted models; but there is a small set of models where one of the methods made a better choice. Therefore, the correlation of the scores for complexes is significantly lower. Most complexes are predicted with acceptable quality, achieving an average DockQ score of 0.53. However, it is important to note that we are only analyzing average DockQ scores, and many of the complexes have multiple interfaces, so some interfaces may still have low quality.

#### 3.1.2 AF3‐Server Versus AlphaFold2

**FIGURE 3:**
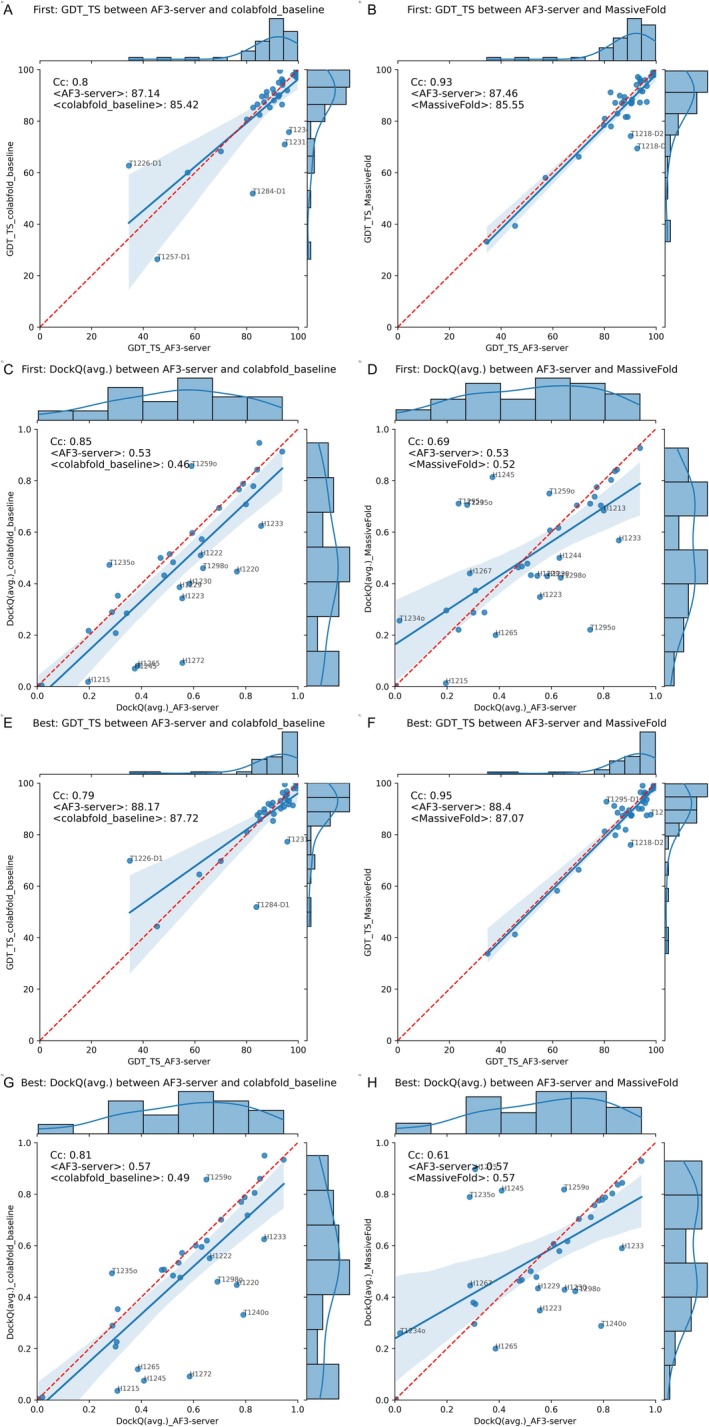
Comparison of AF3‐server with MassiveFold and colabfold_baseline predictions using GDT_TS and DockQ. The top row (A–D) displays the highest‐ranked prediction, while the second row (E–H) highlights the best prediction out of five. The comparison is conducted at the domain level using GDT_TS in the left two columns (A, B, E, and F), and in the right two columns (C, D, G, and H), the comparison for complexes is made using DockQ The comparison against colabfold_baseline is indicated in A, C, E, and G, while the comparison against MassiveFold appears in the other figures. Any target with a difference greater than 10% of the maximum value for one of the methods is annotated with the target number.

Next, we compared the performance of AF3‐server to that of its predecessor, AlphaFold2 (Figure 3). Several versions of what we believe are pure AlphaFold2 predictions are available, including multiple iterations of ColabFold and MassiveFold. Here, we only compared AF3‐server with colabfold_baseline and MassiveFold to determine if the increased sampling used in MassiveFold affected performance. AF3‐server shows a slight advantage among the top‐ranked domains (Figure 3A,B), with an average GDT_TS of 87.1 compared to approximately 85.5 for the AlphaFold2 methods. This difference can be attributed to a few targets where the AF3‐server performed better, although these are not the same targets as those for colabfold_baseline or MassiveFold. The distinction diminishes when examining the best among the five submitted models, see Figure 3E,F. Notably, even when considering the best models, there are a few domains where AF3‐server outperforms the previous version, with one domain exhibiting better results for AlphaFold2.

Regarding complexes, AF3‐server demonstrates an advantage over the baseline established by ColabFold, achieving an average score of 0.53 compared to AlphaFold's 0.46 for first‐ranked models and 0.57 versus 0.49 for the best models, Figure 3C,G,. Approximately 10 models outperform the corresponding models from colabfold_baseline. However, MassiveFold produces models similar in quality to AF3‐server (Figure 3D,H), indicating that increased sampling enables AlphaFold2 to achieve performance comparable to AF3‐server. The unanswered question remains whether increased sampling would have also improved AF3‐server models. In a recent study, we have demonstrated that this is the case for antibody–antigen complexes [24].

#### 3.1.3 Comparison With Top‐Ranked Predictors

**FIGURE 4:**
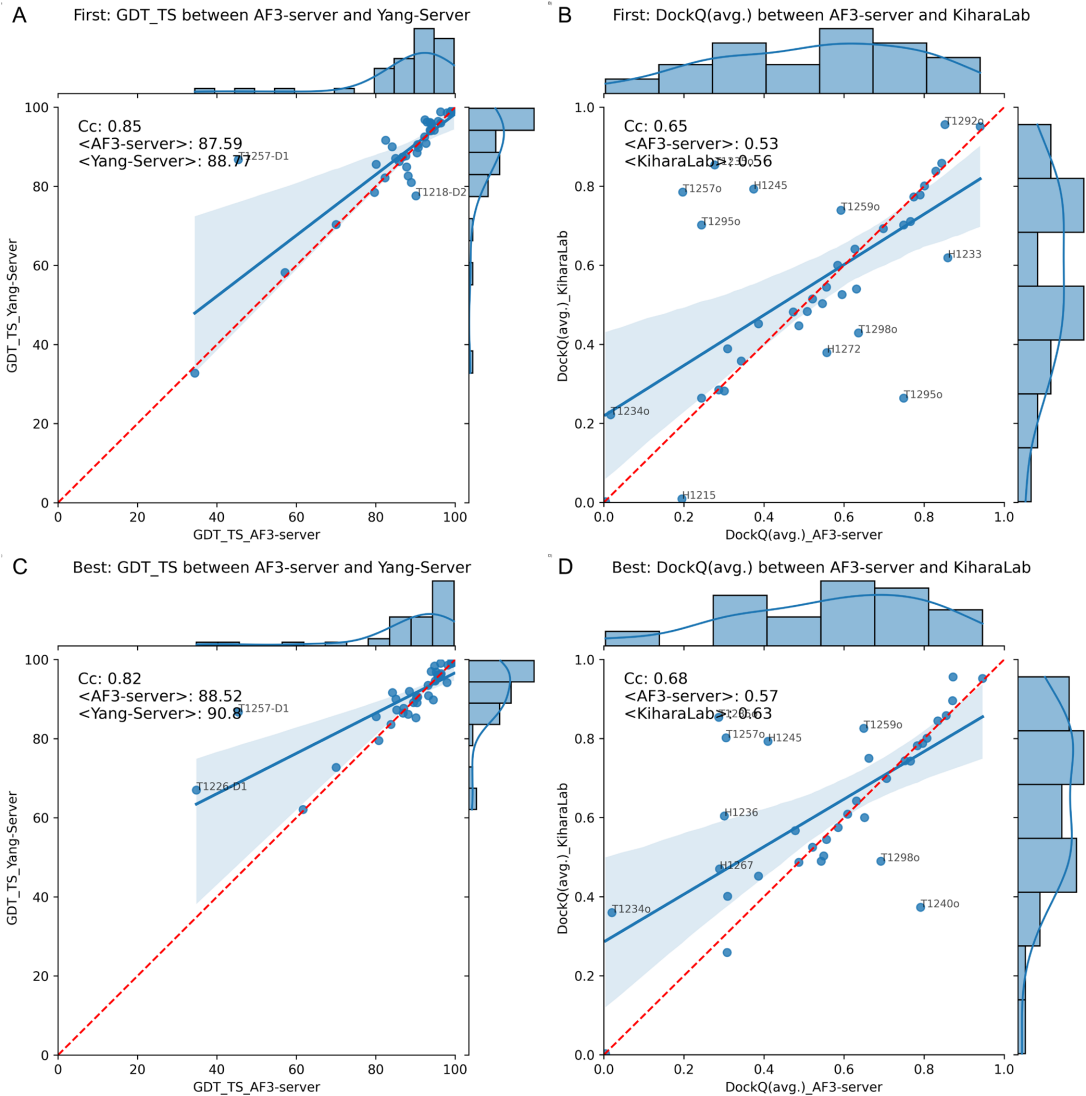
Comparison of AF3‐server and top‐performing predictors, YangServer for domains (A and C) and KiharaLab for complexes (B and D). The top row (A, B) illustrates the highest‐ranked predictions, while the second row (C, D) highlights the best prediction among the five submitted models. On the left, the comparison is conducted at the domain level using GDT_TS, and on the right, the comparison of complexes utilizes DockQ. Any target with a difference greater than 10% of the maximum value for one of the methods is marked with the target number. A rolling average over 10 points is displayed in purple.

Next, we compared AF3‐server performance to that of the top‐ranked predictors. There is always room for discussion regarding which group performed best overall. However, we utilized the prediction center's default settings to select the top groups, resulting in YangServer for domains and KiharaLab for complexes. Our analysis revealed relatively small performance differences among the top‐ranked models for domains (Figure 4A) and complexes (Figure 4B). However, the difference is more pronounced when comparing the best of the five submitted models, as illustrated in Figure 4C,D,. The difference can be attributed almost entirely to two targets (T12561‐D1 and T1226‐D1) for the domains. KiharaLab predicts several complexes better than AF3‐server. However, for two complexes (T1240 and T1298), AF3‐server outperformed KiharaLab.

The results indicate that YangServer and KiharaLab produced better “best” out of five models, but less so among superior top‐ranked models. This might suggest that the top‐ranked methods submitted models with a larger variation than those produced by AF3‐server, similar to how MassiveFold used AlphaFold2. However, methods also struggle to identify the best among the submitted models.

#### 3.1.4 First Versus Best

**FIGURE 5:**
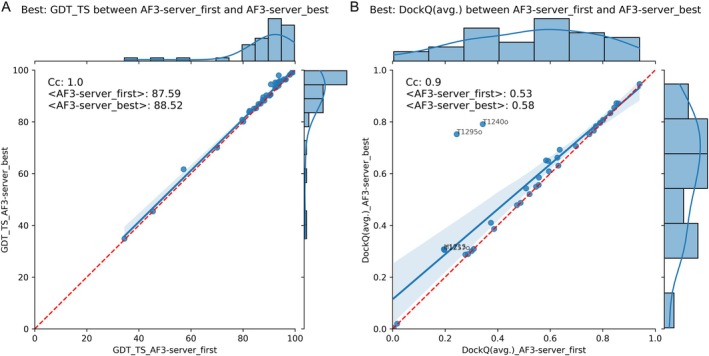
Comparison of the top‐ranked predictions versus the best predictions for each target from the AF3 server. (A) Illustrates domains using GDT_TS, and (B) depicts complexes using DockQ. The target number is annotated for all figures that differ by more than 10%.

In Figure 5, we compare the performance of the top‐ranked model with the best prediction among all AF3‐server predictions. Submitting five models improved the performance for only two complexes: T1240 and T1295. There was no significant improvement for any of the other models. This shows, again, that for hard prediction cases, generating multiple models can help, but the AF3‐server can not always detect the best model.

### 3.2 RNA

**FIGURE 6:**
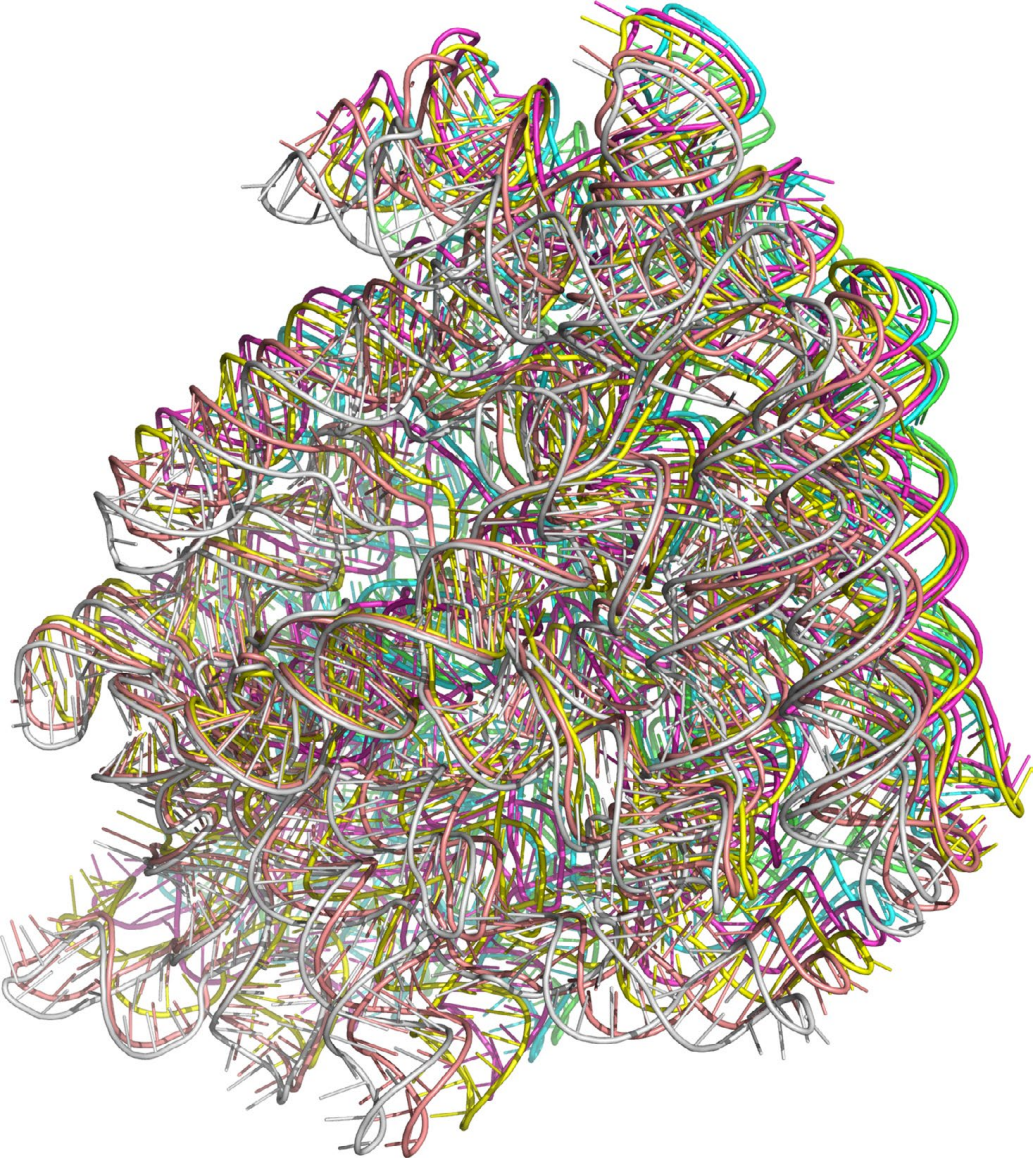
Example of an “overlapping” RNA prediction for R1250 (hexamer), each chain is highlighted in one color. It is clear that they are all superposed on top of each other.

In our analysis of AF3‐server performance, we noted that the software frequently predicted overlapping models for multimeric RNA targets (see Figure 6). As evident from both visual inspection and the scores, we addressed this issue. We reran the predictions until we obtained at least five models without significant overlap and with positive pTM values. In CASP16 official data, the nucleic acid results are categorized into three groups: single RNA, multi‐chain RNA, and hybrid complexes. We merged the latter two categories for our analysis since the mixed category contains only a few entries. However, it should first be noted that there are only a few acceptable models for complexes overall, making significant changes challenging to identify. Further, all three methods predicted the same two complexes with a DockQ larger than 0.6, M1293 and M1296, demonstrating consistency across the methods and highlighting that for M targets, good performance primarily stems from accurate predictions of protein–protein interactions.

**FIGURE 7:**
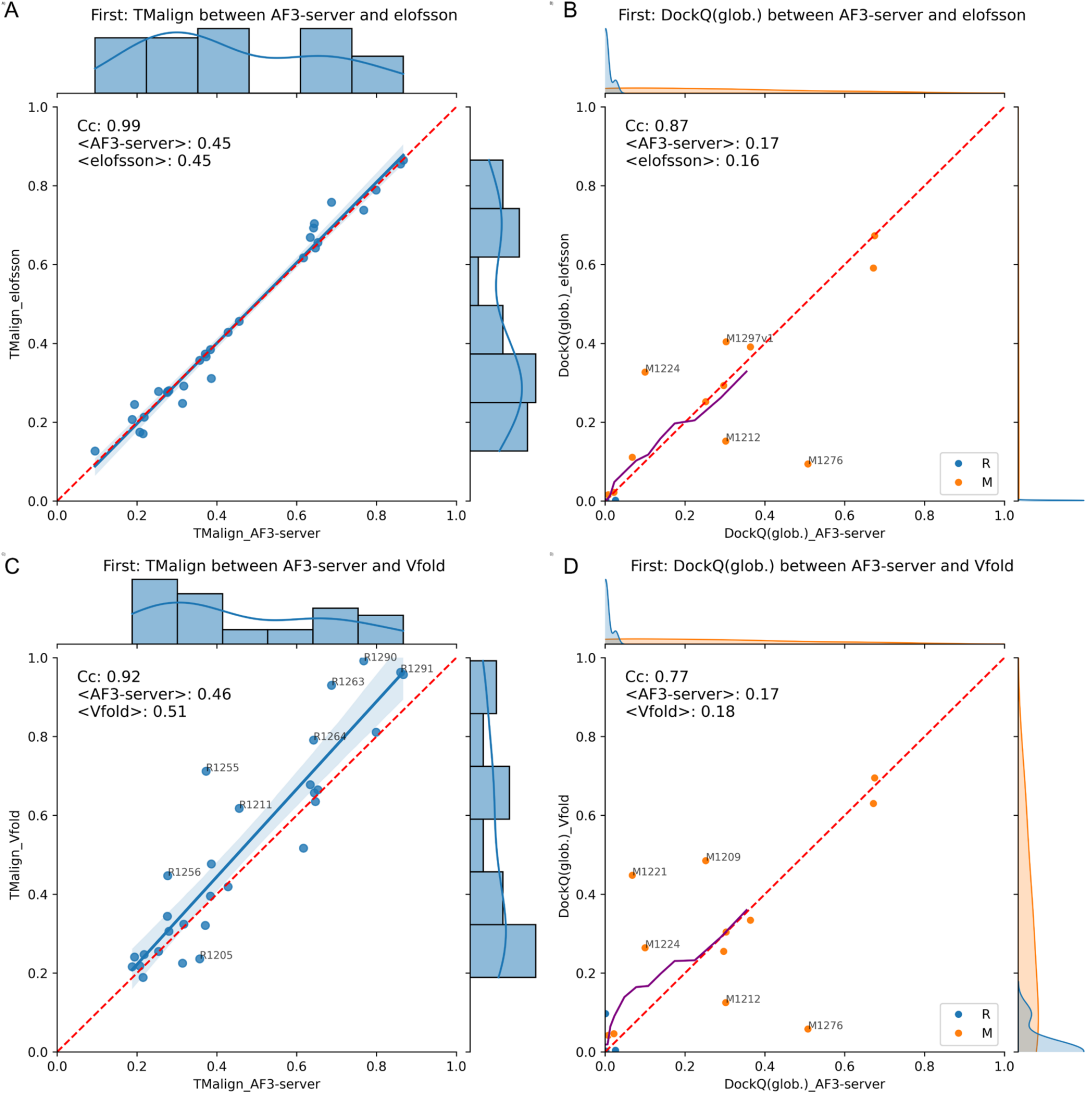
Comparison of RNA predictions. (A) and (B) Compare the AF3‐server with Elofsson predictions, while in (C) and (D) the comparison is between the AF3‐server and the top‐ranked method, Vfold. In (A) and (C), the comparison is made at the single RNA level using TMalign, while in (B) and (D), the comparison of complexes employs DockQ. Here, the Mixed (M) and pure RNA targets are marked in different colors. Any target with a difference greater than 10% of the maximum value for one of the methods is annotated with the target number. A rolling average over 10 points is shown in purple in B and D.

The results show no difference in performance between the Elofsson method and the AF3‐server (see Figure 7A,B). When evaluating single RNA predictions, Vfold [25] slightly outperformed AF3‐server on easier targets, particularly when studying the best of the five submitted models (Figure 7B). Here, the average TMscore for single‐strand RNAs is 0.51 for Vfold compared to 0.46 for AF3‐server. The difference between Vfold and AF3‐server is minimal for complexes, with an average of 0.18 versus 0.17 DockQ. Each method predicts two “M” models significantly better than the other, as shown in Figure 7D.

### 3.3 Estimated Model Accuracy (EMA)

**FIGURE 8:**
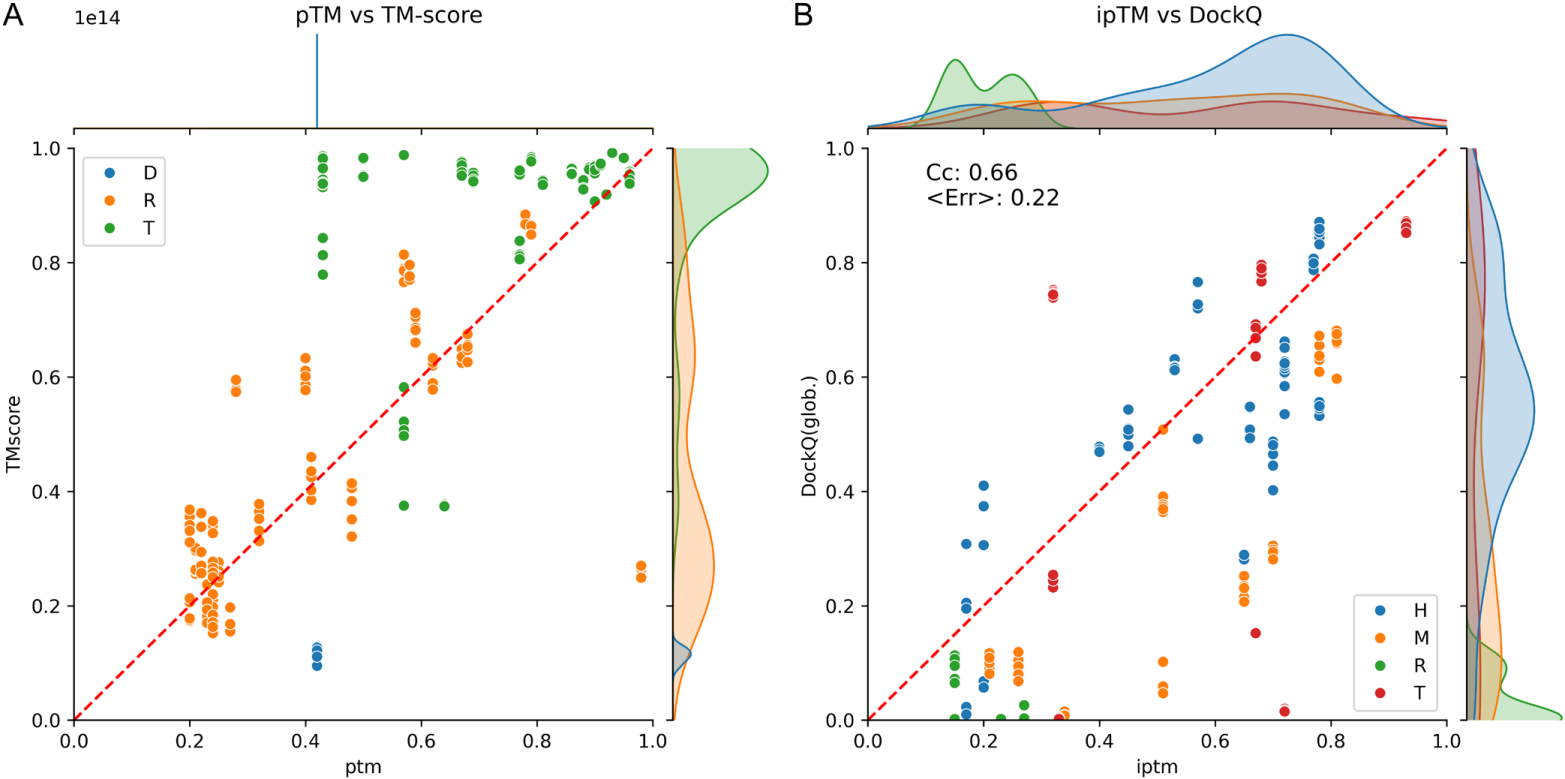
Evaluation of quality estimations. (A) pTM versus TMscore for protein domains and single‐stranded RNA. (B) ipTM versus DockQ for entire complexes. Each complex type (homomeric targets [T], heteromeric targets [H], mixed targets [M], and nucleic acid targets [R]) is represented in different colors.

When evaluating the per‐atom *Z*‐score LDDT RMSD, AF3‐server and Elofsson were ranked second and third, respectively, after PLMfold. For details, see the official assessment on the prediction center. However, we also wanted to compare the predicted TMscores (pTM/ipTM) with the overall quality of a protein/complex. Figure 8A shows that the pTM scores vary significantly between the different protein domains, while almost all domains have a relatively high TMscore; that is, the correlation is not that strong. This is caused by the fact that AF3‐server predicts the pTM for the entire complex, rather than for an individual chain/domain. Therefore, the correlation of prediction accuracy is low (Cc = 0.66). For single‐strand RNA, there seems to be a better agreement of pTM and TM, possibly because these targets were not divided into domains. To provide pTMs for each chain/domain would be a valuable extension to AF3‐server.

For complexes, we compared ipTM with DockQ, as this estimates the quality of the entire complex. Figure 8B shows that the overall correlation is acceptable (*C*_c_ = 0.66); however, if we compare the pTM with the TMscore, the correlation is better (*C*_c_ = 0.83), and the average error is 0.13, data not shown. The good correlation can partly be explained by the fact that almost all RNA models are bad, and the pTM scores are also low.

### 3.4 Stoichiometry

In CASP16, there was a new challenge: to predict the stoichiometry of complexes. We predicted the stoichiometry for 41 stage zero targets, using the AF3‐server scores; see Table 1. Out of the 41 targets, AF3‐server predicted the correct stoichiometry for 14 targets (34%) and 22 (54%) when including all five submitted models. The Elofsson group did slightly better, correctly predicting one additional first‐ranked target and two additional among all submitted targets. The mixed targets (M) were more challenging to submit as both groups only managed to predict one correct stoichiometry for one target (M1287) in the five models submitted. The prediction among all models is better for homomeric (T) targets than for heteromeric targets (H), which is likely explained by the fact that there are many fewer options to generate stoichiometries for homomeric targets. Anyhow, it is clear that AF3‐server cannot reliably predict the stoichiometry for all targets. Still, at least for symmetric homomeric targets, it is often possible to guess them correctly using five models.

**TABLE 1:** The fraction of correctly predicted stoichiometries, by AF3‐server and Elofsson for 41 round zero targets.

	Number	AF3‐server top	AF3‐server best	Elofsson top	Elofsson best
All	41	34%	54%	37%	59%
Heteromeric (H)	19	37%	53%	32%	58%
Mixed (M)	3	0%	33%	0%	33%
RNA (R)	9	33%	44%	44%	56%
Homomeric (T)	10	40%	70%	60%	80%

*Note*: Results are reported for the top‐ranked model or the best of the five submitted models.

## 4 Discussion

There are many ways to evaluate the performance of different groups, and as can be seen above, it is hard to detect the differences using real numbers. Therefore, in CASP, the relative performance, measured by *Z*‐score, is often used to rank the predictions. Using *Z*‐scores highlights minor differences that may have little impact on the normal usage of these methods but help rank them. We did not use the *Z*‐scores above; we assess that they might provide additional information. Therefore, we also compared the ranking of AlphaFold2‐ and AF3‐server‐based methods using the *Z*‐scores from the prediction center. For simplicity, we decided to use the default evaluation by the prediction center and focused only on the groups' rank, using the Sum of *Z*‐scores greater than −2.0. Other measures would have provided similar results, but the details would have varied.

For comparison, we also include colabfold_baseline and MassiveFold in the results, as shown in Table 2. It can be seen that AF3‐server was consistently placed among the top 20% (mainly in the top 10%) of the predictors for all, easy, and medium targets; but only in the top 30%–40% for the hard targets. Also, it is clear that for all except the hard targets, the predictions are significantly better than for Colabfold_baseline or MassiveFold (both based on AlphaFold2), showing that AF3‐server indeed is better than AlphaFold2 for the easier targets. Surprisingly, the performance of AF3‐server for both hard single domain and hard multimer targets seems not to provide any significant improvement over AlphaFold2. It is also clear that massive sampling approaches (see, for instance, the performance of the Wallner group for hard targets) applied to AlphaFold2 provide better results than AF3‐server.

**TABLE 2:** Official rank of the Elofsson (elo) and AF3‐server (Af3) predictors compared with two AlphaFold2‐based methods, colabfold_baseline (Co) and MassiveFold (Ma), at the prediction centers website, using the *Z*‐score > −2 measure.

	All				Easy				Medium				Hard			
Rank	AF3	elo	Ma	Co	AF3	elo	Ma	Co	AF3	elo	Ma	Co	AF3	elo	Ma	Co
Protein Domains (110 predictors)	13	**9**	40	51	**7**	9	36	50	19	**10**	38	54	38	35	50	**25**
Protein Multimers (81 predictors)	**10**	16	30	47	15	**8**	33	25	**2**	16	34	39	35	21	**20**	66
RNA/DNA Monomers (64 predictors)	9	**6**														
RNA/DNA Multimer (32 predictors)	**8**	12														

*Note*: The numbers represent the rank of each predictor. Bold numbers represent the highest ranked method in each category.

## 5 Conclusions

We could not detect any improvement in our manual predictions, Elofsson, over the AF3 server. When comparing AF3‐server to AlphaFold2, AF3‐server performs slightly better for complexes; however, this difference becomes negligible when utilizing massive sampling techniques for AlphaFold2. For domain predictions, no discernible differences were observed between the two versions. Using the official CASP ranking, it was clear that AF3‐server performs relatively better on easier targets, but not harder ones, compared to the other predictors. Moreover, while the best methods, likely incorporating AF3‐server as part of their overall pipeline, are only marginally better than the AF3 server for first‐ranked models, they show improved performance when examining the top model out of five submitted. This suggests that in the context of CASP, it remains beneficial to submit a variety of models to enhance ranking, but also that methods cannot identify the best out of the submitted model. Whether this is useful for applications outside CASP can be discussed, but it highlights that improved selection of the best models would be advantageous. We also show that less than half of the stoichiometries are predicted correctly, indicating an area for future improvement, particularly for non‐symmetric complexes. Regarding RNA predictions, it was observed that most models exhibit low quality, with only a slight difference in performance among the best methods and the AF3‐server, indicating that RNA predictions remain a challenge. For a regular user the default settings of AF3‐server should provide close to state‐of‐the‐art results for all types of targets, but check for potential clashes when submitting multiple chains of nucleic acids.

## 6 Availability

AlphaFold3 was only available as a web server for non‐commercial entities during CASP16. Now it is available in source code from https://github.com/google‐deepmind/alphafold3 under the “Attribution‐NonCommercial‐ShareAlike 4.0 International” license. All analysis scripts are freely available from https://gitlab.com/arneelof/CASP16‐predictions. Details about all predictions can be found here https://github.com/ElofssonLab/casp16/blob/master/Targets.MD.

## Author Contributions

**Arne Elofsson:** conceptualization, investigation, funding acquisition, writing – original draft, writing – review and editing, visualization, validation, methodology, project administration, formal analysis, software, resources, data curation, supervision.

## Conflicts of Interest

The author declares no conflicts of interest.

## Data Availability

The data that support the findings of this study are openly available in Models generated by AlphaFold3 used for CASP16 predictions at https://doi.org/10.1101/2025.04.10.648174, reference number 10.1101/2025.04.10.648174.
